# Brom-hydrate of Quinia

**Published:** 1876-08

**Authors:** 


BROM-HYDRATE OF QUINIA.—Dr. Geo. B. Orr presented to the Cincinnati Academy of Medicine a specimen of this article, and stated that for two weeks he had been testing it in the same doses as he had been accustomed to administer the sulphate. He considered the brom-hydrate twenty times as soluble as the sulphate. Its ready solubility causes it to be quickly absorbed, and its constitutional effects to be more rapidly produced. Hence, in the treatment of intermittent fever, it need not be administered so long before the expected chill.—*The Clinic,* May 13, 1876.

